# Protective Effects of Astragaloside IV against LPS-Induced Endometritis in Mice through Inhibiting Activation of the NF-κB, p38 and JNK Signaling Pathways

**DOI:** 10.3390/molecules24020373

**Published:** 2019-01-21

**Authors:** Fengge Wang, Shuxiong Chen, Liang Deng, Lu Chen, Yuwen Huang, Meng Tian, Chunjin Li, Xu Zhou

**Affiliations:** College of Animal Sciences, Jilin University, Changchun, 5333 Xian Road, Changchun, Jilin 130062, China; wangfg16@mails.jlu.edu.cn (F.W.); chensx14@mails.jlu.edu.cn (S.C.); dengliang17@mails.jlu.edu.cn (L.D.); luchen@jlu.edu.cn (L.C.); huangyw18@mails.jlu.edu.cn (Y.H.); tianmeng18@mails.jlu.edu.cn (M.T.)

**Keywords:** Astragaloside IV, endometritis, LPS, NF-κB, p38, JNK

## Abstract

Endometritis, inflammation of the endometrium, is a common reproductive obstacle disease that can lead to infertility in female animals. Astragaloside IV (AS IV), one of the major and active components of the *Astragalus membranaceus* (Fisch.) Bunge, is known for its anti-inflammatory effects. In the present study, the effects and mechanisms of AS IV on lipopolysaccharide (LPS)-induced endometritis were investigated using a mouse model. Female mice were prepared with AS IV (0.01 mg/g) by gavage for six days before being stimulated with LPS. The results showed that the histopathological changes, levels of inflammatory cytokines (IL-1β and TNF-α), concentration of NO, and myeloperoxidase (MPO) activity in LPS-induced uteri were attenuated significantly by pretreatment with AS IV. Furthermore, LPS-induced activations of NF-κB, p38, and JNK signal pathways were suppressed by pretreatment with AS IV. In conclusion, the data provided new evidence that AS IV effectively attenuates LPS-induced endometritis through inhibition of TLR4-mediated NF-κB, p38, and JNK signaling pathways, implying that AS IV might become a promising potential anti-inflammatory agent for endometritis and other inflammatory diseases.

## 1. Introduction

Endometritis is a common reproductive obstacle disease in female animals which is generally induced by environmental pollutants that lead to the secretion of purulent inflammatory mucus in uterine tissues [1]. Many sterility outcomes may be associated with the presence of this inflammatory response that lead to the inability of the endometrium to maintain viable embryos. It is well known that endometritis decreases productivity in cows, which would trigger huge economic losses for the dairy industry [2]. 

**Figure 1:**
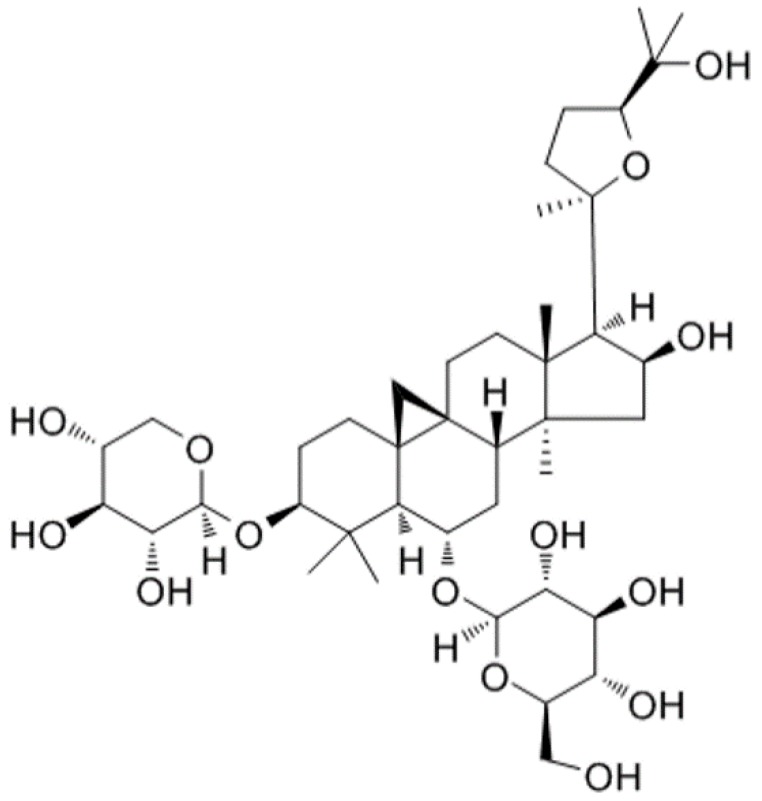
Chemical structure of Astragaloside IV.

Lipopolysaccharide (LPS) is classified as an important component of the outer membrane of Gram-negative bacteria, which is required for viability of most strains [3]. LPS is known for its strong biological activities that can enter the bloodstream and result in inflammatory responses [4]. LPS can activate the relevant receptors on the cell membrane and result in the release of proinflammatory mediators, including tumor necrosis factor-α (TNF-α) and interleukin-1β (IL-1β). Therefore, LPS is widely used to establish mammalian models of inflammation for studying inflammatory disease [5]. For example, a mouse model of endometritis induced by LPS has been made available as a preliminary study disease [6]. Recently, some studies have focused on the treatment of inflammatory diseases such as endometritis and mastitis with herbal medicines [7,8]. Thus, it is important and imperative to explore safe and effective compounds of herbal medicines to treat endometritis. *Astragalus membranaceus* (Fisch.) Bunge is a widely used Chinese medicinal herb that is well-known for its vital-energy tonifying, diuretic, skin reinforcing, tissue generative actions and abscess-draining [9,10,11]. As a key active component of the *Astragalus membranaceus* (Fisch.) Bunge, Astragaloside IV (AS IV) (Figure 1) possesses pharmacological effects such as anti-inflammatory [12], anti-diabetes [13], and anti-hypertension [14] effects. Evidence from pharmacological studies and clinical practice has shown that AS IV has been shown to have a powerful therapeutic effect on many diseases, including kidney injury [15], nephritis [16], hepatitis [17], diabetes [18], and heart failure [19], by removing free radicals and decreasing lipid peroxidation. However, the anti-inflammatory role of AS IV on LPS-induced endometritis has not been studied. The purpose of the present study was to investigate the protective effects and mechanisms of AS IV on LPS-induced endometritis. First, we established an endometritis mice model with LPS after pretreatment with AS IV. Second, we focused on the protective effects of AS IV against LPS-induced endometritis concerning the aspects of histopathological changes, inflammatory cytokine levels (IL-1β and TNF-α), concentration of NO, and myeloperoxidase activity in the uterus. Third, we investigated the protective mechanisms in the aspects of NF-κB, p38, and JNK signaling pathways.

## 2. Results

### 2.1. Effect of AS IV on LPS-Induced Histopathological Changes of Uterus

**Figure 2:**
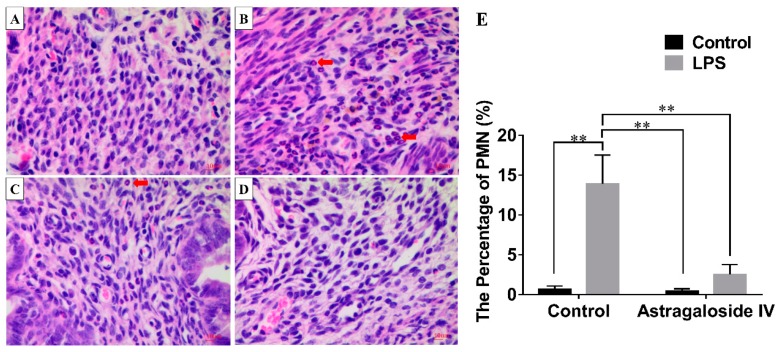
Effects of Astragaloside IV (AS IV) on lipopolysaccharide (LPS)-induced histopathological changes of uteri (hematoxylin-eosin staining (HE), ×400). Mice uteri from each group were processed for histological evaluation at 24 h after LPS infusion. (**a**): LPS^−^/AS IV^−^ group. (**b**): LPS^+^/AS IV^−^. (**c**): LPS^+^/AS IV^+^ group. (**d**): LPS^−^/AS IV^+^ group. Red arrows represent polymorphonuclear neutrophils. (**e**): The percentage of polymorphonuclear neutrophils (PMN). Two-way analysis of variance was performed with a Bonferroni post hoc multiple comparison test. The data are shown as mean ± standard deviation (SD). *n* = 10. ** *p* < 0.01.

**Figure 3:**
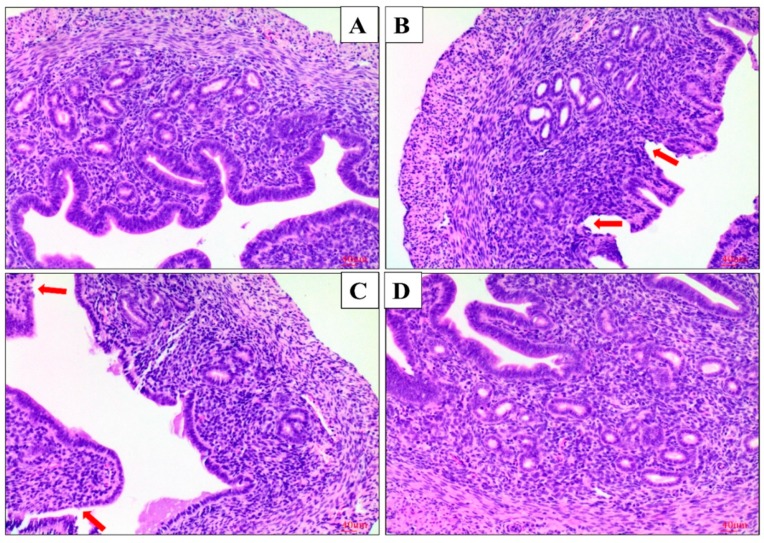
Effects of Astragaloside IV on LPS-induced histopathological changes of uteri (HE, ×200). Mice uteri from each group (*n* = 15) were processed for histological evaluation at 24 h after LPS infusion. The shedding of epithelial cells and the destroyed integrity of the endometrium in the endometritis group were observed. Red arrows represent the imperfection of the endometrium and the shedding of epithelial cells. (**a**): LPS^−^/AS IV^−^ group. (**b**): LPS^+^/AS IV^−^ group. (**c**): LPS^+^/AS IV^+^ group. (**d**): LPS^−^/AS IV^+^ group.

The morphological changes of uteri were observed with a microscope. There was no pathological change in the control (LPS^−^/AS IV^−^) group. In the LPS^+^/AS IV^−^ group the uterus tissues were markedly damaged, with hyperemia, hemorrhages, many polymorphonuclear neutrophils (PMN) infiltrating the uteri, the shedding of epithelial cells, and damaged endometria (Figure 2 and Figure 3) being exhibited. However, with pretreatment with AS IV (the LPS^+^/AS IV^+^ group), the LPS-induced histopathologic changes were significantly attenuated (Figure 2 and Figure 3). The cell number of PMN in the LPS^+^/AS IV^−^ group was significantly greater than that in the LPS^+^/AS IV^+^ group. AS IV (for the LPS^+^/AS IV^+^ group) significantly decreased the cell number of PMN compared with the LPS^+^/AS IV^−^ group (Figure 2e). Two-way analysis of variance (ANOVA) revealed a main effect of LPS (*F* = 108.33; *p* < 0.01) and AS IV (*F* = 44.57; *p* < 0.01), and an interaction effect of LPS × AS IV (*F* = 42.17; *p* < 0.01). Post hoc analysis showed that LPS significantly increased the cell number of PMN compared with the control group (*p* < 0.01), and that AS IV treatment in the presence of LPS significantly reduced the cell number of PMN compared with LPS alone (*p* < 0.01) (Figure 2e).

### 2.2. Effect of AS IV on Myeloperoxidase (MPO) Activity in LPS-Induced Uterus

**Figure 4:**
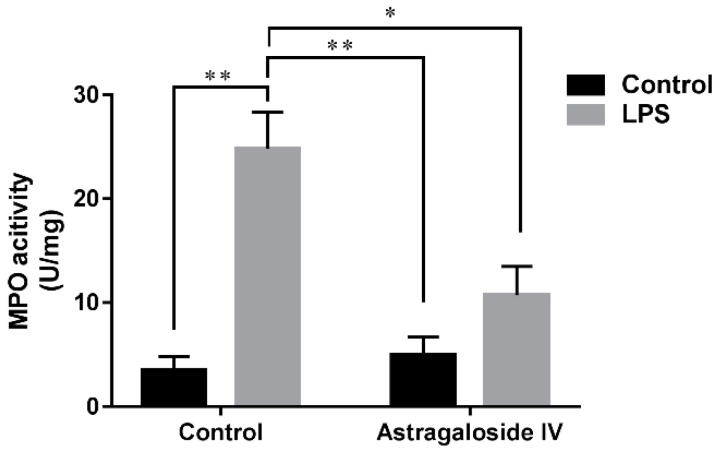
Effects of Astragaloside IV on myeloperoxidase (MPO) activity of LPS-induced uteri. MPO activity was determined at 24 h after LPS infusion. Two-way analysis of variance was performed with a Bonferroni post hoc multiple comparison test. Data are shown as mean ± SD. *n* = 6. * *p* < 0.05; ** *p* < 0.01.

Increased MPO activity is one of early markers of inflammatory diseases, reflecting the level of oxidative stress and inflammation [20]. The results of the present study showed that the MPO activity of the LPS^+^/AS IV^−^ group was significantly increased compared to that of the LPS^−^/AS IV^−^ group, while AS IV (for the LPS^+^/AS IV^+^ group) significantly decreased MPO activity compared to activity in the LPS^+^/AS IV^−^ group (Figure 4). Two-way ANOVA revealed a main effect of LPS (*F* = 28.38; *p* < 0.01) and AS IV (*F* = 6.19; *p* < 0.05), and an interaction effect of LPS × AS IV (*F* = 9.37; *p* < 0.05). Post hoc analysis showed that LPS significantly increased MPO activity compared with the control group (*p* < 0.01), and that AS IV treatment in the presence of LPS reduced MPO activity compared with LPS alone (*p* < 0.05) (Figure 4).

### 2.3. Effect of AS IV on Concentration of NO in LPS-Induced Uteri

**Figure 5:**
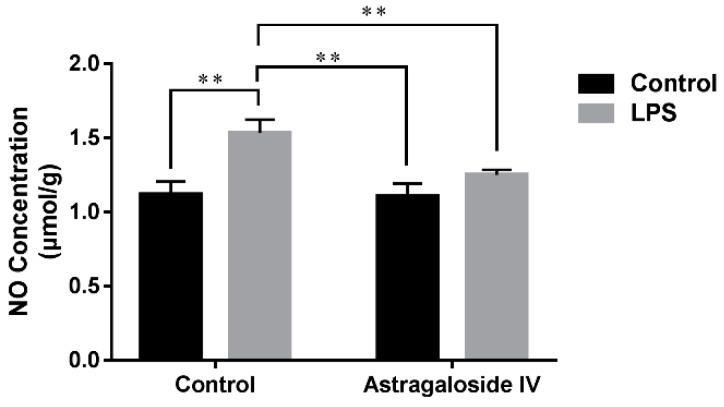
Effects of Astragaloside IV on the concentration of NO in LPS-induced uteri. The concentration of NO was determined at 24 h after LPS infusion. Two-way analysis of variance was performed with a Bonferroni post hoc multiple comparison test. Data are shown as mean ± SD. *n* = 6. **, *p* < 0.01.

Increased concentration of NO is another early marker of inflammatory diseases which reflects levels of oxidative stress and inflammation [21]. As shown in Figure 5, the concentration of NO in the LPS^+^/AS IV^−^ group was significantly greater than that in the LPS^−^/AS IV^−^ group, while AS IV (for the LPS^+^/AS IV^+^ group) significantly decreased NO concentration compared to the LPS^+^/AS IV^−^ group (Figure 5). Two-way ANOVA revealed a main effect of LPS (*F* = 14.14; *p* < 0.01) and AS IV (*F* = 51.36; *p* < 0.01), and an interaction effect of LPS × AS IV (*F* = 12.27; *p* < 0.01). Post hoc analysis showed that LPS significantly increased the concentration of NO compared with the control group (*p* < 0.01), and that AS IV treatment in the presence of LPS significantly reduced the concentration of NO compared with LPS alone (*p* < 0.01) (Figure 5).

### 2.4. Effect of AS IV on LPS-Induced IL-1β and TNF-α Production in Mouse Uteri

**Figure 6:**
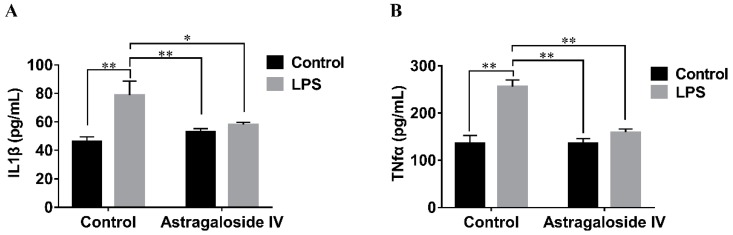
Effects of Astragaloside IV on the production of IL-1β and TNF-α in uteri. The levels of IL-1β and TNF-α were detected by ELISA assay. Two-way analysis of variance was performed with a Bonferroni post hoc multiple comparison test. Data are shown as mean ± SD. *n* = 6. * *p* < 0.05; ** *p* < 0.01.

The effects of AS IV on LPS-induced IL-1β and TNF-α production in mouse uteri were detected by ELISA assay. Stimulation with LPS led to a significant increase in IL-1β and TNF-α production compared to the LPS^−^/AS IV^−^ group, but AS IV (for the LPS^+^/AS IV^+^ group) decreased the production of IL-1β and TNF-α compared to the LPS^+^/AS IV^−^ group (Figure 6). With regard to IL-1β production, two-way ANOVA revealed a main effect of LPS (*F* = 19.59; *p* < 0.01) but not of AS IV (*F* = 2.50; *p* > 0.05), as well as an interaction effect of LPS × AS IV (*F* = 10.40; *p* < 0.01). Post hoc analysis showed that LPS significantly increased IL-1β production compared with the control group (*p* < 0.01), and that AS IV treatment in the presence of LPS reduced IL-1β production compared with LPS alone (*p* < 0.05) (Figure 6a). With regard to TNF-α production, two-way ANOVA revealed a main effect of LPS (*F* = 160.13; *p* < 0.01) and AS IV (*F* = 71.74; *p* < 0.01), and an interaction effect of LPS × AS IV (*F* = 73.66; *p* < 0.01). Post hoc analysis showed that LPS significantly increased TNF-α production compared with the control group (*p* < 0.01), and that AS IV treatment in the presence of LPS significantly reduced TNF-α production compared with LPS alone (*p* < 0.01) (Figure 6b).

### 2.5. AS IV on LPS-Induced Activation of TLR4 and the NF-κB Signal Pathway

**Figure 7:**
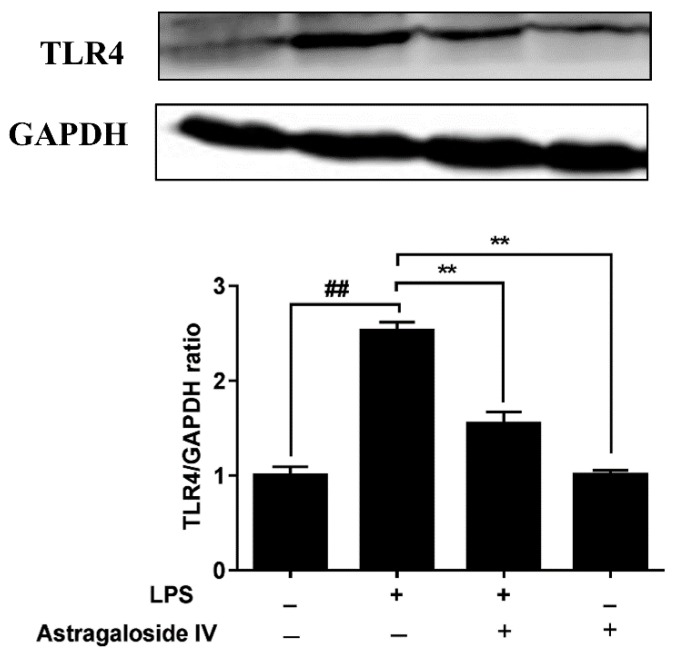
Effects of AS IV on LPS-induced TLR4 protein expression in uteri. Protein levels were detected by Western blotting. Relative protein levels were analyzed by grey scanning. The data are shown as mean ± SD, *n* = 3; ** *p* < 0.01. ## indicates a significant difference from the LPS^−^/AS IV^−^ group (*p* < 0.01).

**Figure 8:**
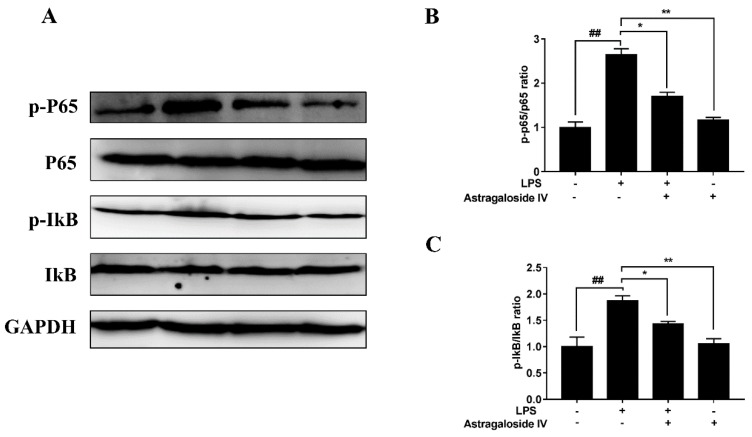
Effects of Astragaloside IV on LPS-induced activation of NF-κB signal pathways in uteri. Protein levels were detected by Western blotting. Relative protein levels were analyzed by grey scanning. The data are shown as mean ± SD, *n* = 3; * *p* < 0.05; ** *p* < 0.01. ## indicates a significant difference from the LPS^−^/AS IV^−^ group (*p* < 0.01).

The NF-κB signal pathway is essential to the regulation of inflammatory responses [22]. TLR4 recognition of LPS is related to the NF-κB signal pathway [23]. In the present study, the protein level of TLR4 was significantly increased in the LPS^+^/AS IV^−^group compared to the LPS^−^/AS IV^−^ group. The TLR4 protein level of the LPS^+^/AS IV^+^ group was significantly decreased compared to that in the LPS^+^/AS IV^−^ group (Figure 7). Furthermore, LPS induced the activation of the NF-κB signal pathway (Figure 8). In addition, the degradation of IκBα and the activation of NF-κB were significantly reserved by AS IV (in the LPS^+^/AS IV^+^ group) (Figure 8).

### 2.6. AS IV on LPS-Induced Activation of p38 and JNK Signal Pathways

**Figure 9:**
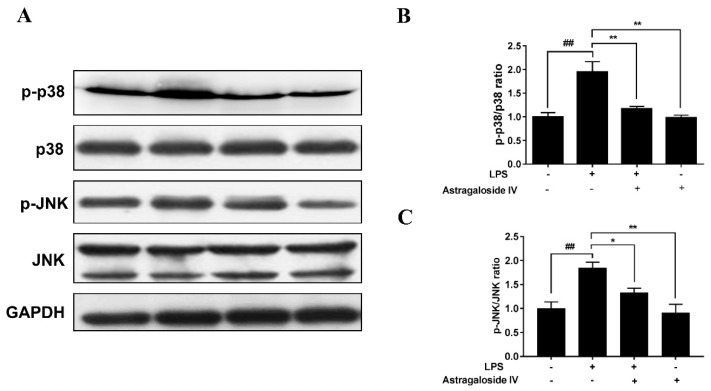
Effects of astragaloside IV on LPS-induced activation of p38 and JNK signal pathways in uteri. Proteins levels were detected by Western blotting. Relative protein levels were analyzed by grey scanning. The data are shown as mean ± SD, *n* = 3; * *p* < 0.05; ** *p* < 0.01. ## indicates a significant difference from the LPS^−^/AS IV^−^ group (*p* < 0.01).

It is well known that p38 and JNK signal pathways are vital signal pathways that regulate inflammatory response [23,24,25]. In the present study, LPS significantly increased the phosphorylation of p38 and JNK compared to the LPS^−^/AS IV^−^ group. However, AS IV (for the LPS^+^/AS IV^+^ group) decreased the phosphorylation levels of p38 and JNK compared to the LPS^+^/AS IV^−^ group (Figure 9).

## 3. Discussion

Endometritis is one of the common diseases for cows in the postpartum period [26], which has caused enormous economic losses for dairy farms. It has been demonstrated that LPS could be used to establish mouse endometritis models as tools to study the disease [27]. In the present study, the protective role of AS IV on endometritis was investigated with an LPS-induced endometritis model. AS IV significantly attenuated histopathological changes and decreased MPO activity, the production of inflammatory cytokines, and concentration of NO in uteri induced by LPS. These results imply a possibility of curative usage of AS IV for the treatment of endometritis.

LPS is widely used to induce the secretion of inflammatory cytokines in tissues. In addition, the histopathological finding has been regarded as the most visual way to determine the obvious inflammatory changes of uterine organization structures [28]. In the present study, we found that the uterine structures of LPS-treated mice were more severely damaged with the shedding of epithelial cells and the infiltration of large numbers of inflammatory cells, suggesting that LPS induced neutrophil accumulation in the uterine tissues. However, the damaged uterine structures were rarely observed with treatment with AS IV, suggesting that AS IV significantly alleviated the uterine damage induced by LPS.

MPO activity is a major biomarker of neutrophils that directly reflects the number of neutrophils in uterine tissue [20]. High concentrations of NO could result in increases in the amount of mononuclear cells and inflammation-based permeability, and cause vasodilatation at the site of inflammation [21]. In the present study, the concentration of NO and MPO activity in uteri induced by LPS were decreased significantly by AS IV pretreatment. These results are consistent with the observation of pathology in uteri.

Evidence suggests that the overproduction of proinflammatory cytokines can be used as a diagnostic biomarker of endometritis [29]. TNF-α is the earliest and key endogenous mediator that takes part in the process of an inflammatory reaction [30]. TNF-α is highly virulent to the uterus that could induce the apoptosis of epithelial cells [31]. In the present study, the higher expression of TNF-α may induce cell apoptosis and result in the shedding of epithelial cells in the uterus. AS IV may attenuate LPS-induced epithelial cells apoptosis through inhibiting the expression of TNF-α. IL-1β plays a key role in the progress of the synthesis of prostaglandin and fibrinogen [32]. Therefore, the potential therapeutic strategy for inflammatory diseases should be the attenuation of pro-inflammatory cytokines. It has been reported that suppressing the expression of IL-1β and TNF-α could attenuate LPS-induced endometritis [33]. In the present study, AS IV inhibited LPS-induced production of IL-1β and TNF-α, meaning that AS IV could attenuate LPS-induced endometritis by inhibiting these inflammatory cytokines.

Recent studies have reported that TLR4 recognition of LPS is related to the NF-κB signal pathway [27,28,34]. LPS activates the NF-κB signal pathway that regulates the release of pro-inflammatory cytokines [22,35]. Furthermore, it has been observed that NF-κB is a regulator of the production of various inflammatory cytokines [22]. Once motivated by LPS, NF-κB is activated by phosphorylation and regulates the production of inflammatory cytokines [23]. Our results showed that the expression of TLR4 and the phosphorylation levels of IκBα and NF-κB were significantly increased with LPS treatment compared to the control group, suggesting that LPS may induce the activation of the TLR4-NF-κB signaling pathway in the uterine tissues of mice with endometritis. Suppression of LPS-induced activation of NF-κB by AS IV suggests that AS IV alleviation of LPS-induced endometritis may be achieved by blocking the NF-κB signaling pathway.

Furthermore, p38 and JNK are the major effectors of the MAPK pathway. The phosphorylation of p38 and JNK proteins can cause the translocation of Activator Protein-1 to the nucleus and promote an inflammatory response [24]. Recent studies have also reported that TLR4 recognition of LPS is related to the MAPK signal pathway [27,28]. In the present study, the phosphorylation levels of p38 and JNK were increased with LPS in mice. In addition, suppression of LPS-induced activation of p38 and JNK by AS IV suggests that AS IV alleviation of LPS-induced endometritis may be achieved by blocking the TLR4-mediated p38 and JNK signaling pathways.

## 4. Materials and Methods

### 4.1. Chemical and Animal Experiments

Female BALB/c mice who were eight weeks of age and weighed about 30 g were bought from the Center of Experimental Animals of Baiqiuen Medical College of Jilin University. To determine if the stages of the estrus cycle affect the diagnostic biomarkers of endometritis detected in the present study, the production of IL-1β and TNFα, NO concentration, and the activity of MPO of uterine tissues from different stages of the estrus cycle (proestrus stage, estrus stage, metestrus stage, and diestrus stage, with eight mice for each stage) were detected. The results showed that all of these parameters were not significantly different among the different stages of the estrus cycle (Figure S1). Therefore, the mice in the subsequent experiments were randomly assigned to different groups. A total of 60 mice were cafeteria feeding and maintained at a 12 h light/dark cycle room. The investigation conformed to the Guide for the Care and Use of Laboratory Animals by NIH (ethics approval number 20150905), and all animal procedures were reviewed and approved by the Jilin University Institutional Animal Care and Use Committee. AS IV (300 mg; Yuanye Biomart, Shanghai, China) was dissolved in propylene glycol (1 mL; Sigma Aldrich, St. Louis, MO, USA) and further diluted with sodium chloride (333 mL; DB, Changchun, Jilin, China). LPS (serotype 055:B5 from *Escherichia coli*, Sigma Aldrich, St. Louis, MO, USA) was dissolved in sodium chloride. The mice were randomly assigned to four groups as follows: (1) the control group (the LPS^−^/AS IV^−^ group) in which mice received the solvents propylene glycol with sodium chloride (0.5 mL) without AS IV and LPS by gavage for six days followed by a single intrauterine perfusion of sodium chloride (25 μL); (2) the LPS-treated group (the LPS^+^/AS IV^−^ group) in which mice received the solvents propylene glycol with sodium chloride (0.5 mL) by gavage for six days followed by an intrauterine perfusion of LPS (2.5 mg/mL, 25 μL); (3) the AS IV plus LPS-treated group (the LPS^+^/AS IV^+^ group) in which mice received AS IV (0.01 mg/g, 0.5 mL) by gavage for six days followed by an intrauterine perfusion of LPS (2.5 mg/mL, 25 μL); (4) the AS IV only group (the LPS^−^/AS IV^+^ group) in which mice received AS IV (0.01 mg/g, 0.5 mL) by gavage for six days followed by an intrauterine perfusion of sodium chloride (25 μL). Mice were sacrificed 24 h after the intrauterine perfusion. The same uteri for each of the four groups were used for histological analysis and the assays.

### 4.2. Histological Assay

The uteri were transferred to 4% paraformaldehyde for 48 h. After this, the uteri were embedded in paraffin. Then, the uterine tissues were cut into 5 μm slices. The sections of uterine tissues were stained with hematoxylin and eosin staining. Subsequently, the sections were observed with a microscope (Olympus, IX71, Tokyo, Japan).

### 4.3. MPO Assay

The homogenates of uterine tissues were prepared on ice. MPO activity in the supernatant was detected using a commercial kit (Nanjing Jiancheng Bioengineering Institute, Nanjing, China) consistent with the manufacturer’s instructions. Uterine tissues of 100 mg were homogenized and fluidized in extraction buffer to obtain 5% of homogenate. The samples, including 0.1 mL of reaction buffer and 0.9 mL homogenate, were heated to 37 °C in water for 10 min, upon which enzymatic activity was determined with a spectrophotometer at 460 nm.

### 4.4. NO Assay

The supernatants of uterine tissues were collected and the concentration of NO in the supernatant was detected using a commercial kit (Solarbio Life Science, Beijing, China) in accordance with the manufacturer’s instructions. NO is easily oxidized to form NO_2_^−^ in vivo or in aqueous solution. The accumulation of NO_2_^−^ was determined as an indicator of NO production in the medium. The product has a characteristic absorption peak at 550 nm and the light absorption value can be used to determine and calculate NO content. Uterine tissues of 100 mg were homogenized and fluidized in extraction buffer to obtain 5% of homogenate. The absorbance was read at 550 nm by using a microplate reader (BioTek, Highland Park, IL, USA).

### 4.5. Enzyme-Linked Immunosorbent Assays

The uteri were weighed and homogenized on ice. Uterine tissues of 100 mg were homogenized. The homogenates were then centrifuged at 12,000 g for 10 min at 4 °C (Fresco 21, Thermo Fisher Scientific Inc., Rockford, Illinois, USA) and the supernatants were collected. The levels of IL-1β and TNFα in the supernatants were detected using an enzyme-linked immunosorbent assay (ELISA; eBioscience, San Diego, CA, USA). These procedures were performed in accordance with the manufacturer’s instructions. The results were detected with a microplate absorbance reader at 450 nm.

### 4.6. Western Blot Analysis

Concentrations of proteins in uterine tissues were detected via a BCA protein assay kit (Beyotime, Shanghai, China). Equal amounts of proteins (40 µg) were loaded in each well and separated by 10% SDS-PAGE and blocked with 5% bovine serum albumin dissolved in TBST for 1 h, and then incubated with primary antibodies (1:1000; Cell Signaling Technology, Beverly, MA, USA; JNK CST9252; p-JNK CST9255; p38 CST8690P; p-p38 CST4511; p65 CST8242; p-p65 CST3033; IκBα CST 4812; p-IκBα CST 2859; TLR4 CST14358; GAPDH CST5174) at 4 °C for 12 h. The membranes were washed four times for 6 min and incubated with the appropriate second antibody conjugates (Abcam, Cambridge, MA, USA) or horseradish peroxidase-conjugated protein antibody for 1 h at room temperature. Then, the membranes were washed four times and stained with DAB Horseradish Peroxidase (Color Development Kit Beyotime, Shanghai, China). The proteins were detected by using the gel visualize Alpha Innotech (Tanon-5200Multi, Tanon Science & Technology Co., Ltd., Shanghai, China). Protein levels were normalized to glyceraldehyde-3-phosphate dehydrogenase (GAPDH) and quantified via densitometry by using a Tanon gel imaging system (Tanon, Shanghai, China).

### 4.7. Statistical Analysis

All data were analyzed with SPSS 22.0 software (Version X, IBM, Armonk, NY, USA). The premise of one-way or two-way ANOVA is that all groups of data obey normal distribution and the variance of each group of data is homogeneous. A one-sample K-S test was used to analyze the normality of the data and *p* > 0.05 was used to indicate that the data conformed to the normal distribution. Levene’s test method was used for the homogeneity of variance test, and *p* > 0.05 was used to indicate that the variance was homogeneous. Then, one-way or two-way analysis of ANOVA with multiple comparison post-tests (Dunnett or Bonferroni) were used to compare the means between experimental groups as indicated. Data have been given as mean ± standard deviation (SD). *p*-Values of 0.05 or less were considered to be significant.

## 5. Conclusions

**Figure 10:**
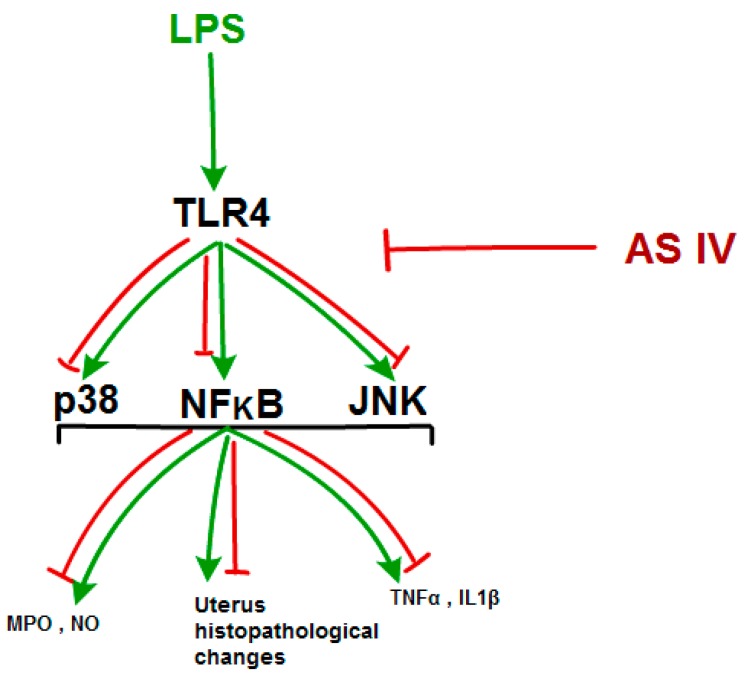
A putative mechanism of Astragaloside IV alleviating LPS-induced endometritis in mice. Green arrows indicate the effects of LPS on the uterus and red arrows indicate the protective effects of Astragaloside IV on uteri containing LPS-induced endometritis.

In summary, this study provided strong evidence of the protective effects of AS IV against LPS-induced endometritis in mice, the mechanisms of which could be deduced as involving AS IV inhibiting the LPS-induced production of IL-1β and TNF-α, the concentration of NO, and MPO activity in uteri through the suppressing of TLR4 mediated NF-κB, p38, and JNK signaling pathways (Figure 10). The result of the present study implies the potential prospects of AS IV for the treatment of endometritis.

## Supplementary Materials

The following are available online. Figure S1: Production of TNFα (**a**) and IL-1β (**b**), the activity of MPO (**c**), and NO concentration (**d**) of uterine tissues of mice in different stages (proestrus stage, estrus stage, metestrus stage and diestrus stage) of the estrus cycle. The data are shown as mean ± SD, *n* = 8.

## Abbreviations

AS IV	Astragaloside IV
MPO	Myeloperoxidase
LPS	Lipopolysaccharide
