# Supplementary material for: Protective Effects of Astragaloside IV against LPS-Induced Endometritis in Mice through Inhibiting Activation of the NF-κB, p38 and JNK Signaling Pathways
Source: Molecules. 2019 Jan 21;24(2):373. doi: 10.3390/molecules24020373 (PMC6360020; doi:10.3390/molecules24020373)
Supplement: Supplementary file 1 [file molecules-24-00373-s001.pdf]

Supplementary Material

Figure S1

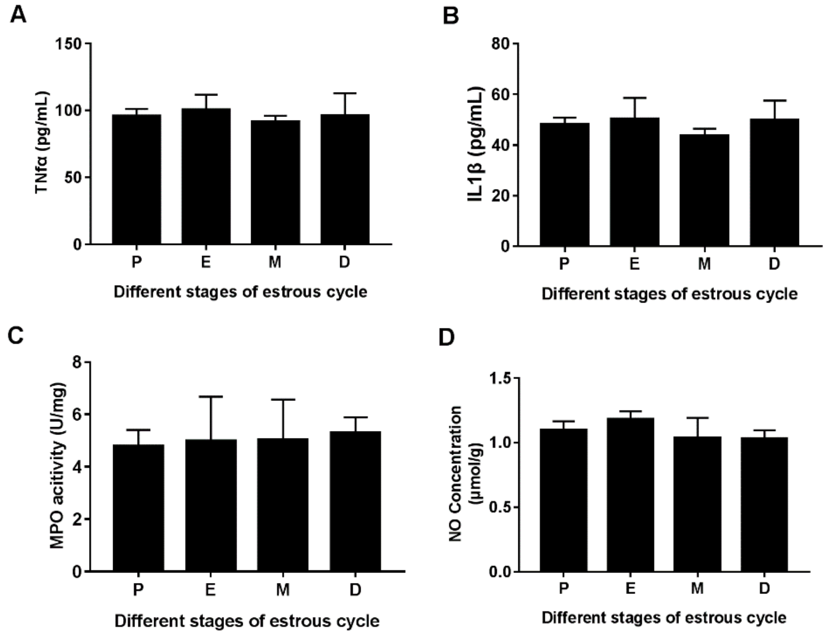

Figure S1. Production of TNFα (**a**) and IL-1β (**b**), the activity of MPO (**c**), and NO concentration (**d**) of uterine tissues of mice in different stages (proestrus stage, estrus stage, metestrus stage and diestrus stage) of the estrus cycle. The data are shown as mean ± SD, n = 8.
